# Clinical Phenotype and Mechanisms of Leukopenia/Neutropenia in Patients with Primary Sjögren’s Syndrome

**DOI:** 10.31138/mjr.33.1.99

**Published:** 2022-03-31

**Authors:** Ioanna E. Stergiou, Efstathia E. Kapsogeorgou, Athanasios G. Tzioufas, Michalis Voulgarelis, Andreas V. Goules

**Affiliations:** Department of Pathophysiology, School of Medicine, National and Kapodistrian University of Athens, Athens, Greece

**Keywords:** Sjögren’s syndrome, leukopenia, neutropenia, clinical phenotype, pathogenesis

## Abstract

Sjögren’s syndrome (SS) is a chronic, systemic autoimmune disease which afflicts mainly the exocrine salivary and lachrymal glands, leading to mouth and eye dryness. However, any organ can be affected during the disease course, resulting in a variety of clinical manifestations. Sjögren’s syndrome clinical manifestations can be classified into glandular (sicca manifestations or parotid swelling), extra-glandular, either nonspecific (arthralgias, arthritis, Raynaud’s phenomenon, fatigue) or peri-epithelial (primary biliary cirrhosis, interstitial nephritis, bronchiolitis), and extra-epithelial (palpable glomerulonephritis, peripheral neuropathy, purpura). In addition, SS patients display high risk for B cell lymphomas due to chronic antigenic stimulation. Although disease pathogenesis remains unclear, genetic, environmental, and immunologic factors are implicated. In the context of systemic autoimmune manifestations, SS patients may also present with hematologic abnormalities including anaemia, leukopenia (mainly neutropenia or lymphopenia), and thrombocytopenia. Although leukopenia has been reported as a laboratory finding in many case series or cohorts of SS patients and in very few studies it has been proposed as an independent risk factor for lymphoma, the clinical phenotype of SS patients with leukopenia/neutropenia and the implicated pathogenetic mechanisms have not been elucidated. In the current study, we intend to analyse the clinical phenotype of leukopenic/neutropenic SS patients and explore the possible pathogenetic mechanisms by detecting anti-neutrophil antibodies and investigate the role of apoptotic pathways, especially the contribution of TRAIL pathway and the cFLIP molecule.

## BACKGROUND/AIM

Sjögren’s syndrome (SS) is a chronic, systemic, autoimmune disorder which cause tissue damage of the epithelial structures of the salivary and lachrymal glands, leading to dry mouth and eyes, respectively.^1–3^ The prevalence of SS is estimated to be 0.1% of the general population, affecting mainly middle-aged women, in a female-to-male ratio of 15:1. Although in the majority of SS patients, clinical manifestations are restricted to those of mild dryness exocrinopathy including oral and ocular dryness, SS may also involve other organs such as the kidneys, the lungs, the skin, the peripheral nerves, and the liver. Approximately 5% of SS patients are expected to develop lymphomas of B cell origin, displaying 7–19 increased fold risk of lymphoma compared to the general population.^1,2^

The main histopathologic feature of SS is the lymphocytic-rich inflammatory infiltrate around the ductal epithelium of the salivary glands which appears to mediate local tissue injury producing dryness.^1^ Similar histopathologic lesions can be observed in other organs/tissues, including the biliary ducts (biliary epithelium), the kidney (tubular epithelium), and the lungs (bronchiolar epithelium), leading to primary biliary cirrhosis, interstitial nephritis and bronchiolitis, respectively. Apart from the typical peri-epithelial lymphocytic infiltration, B cell hyper-activity is another important immunopathologic hallmark of SS associated with hypergammaglobulinemia and production of several autoantibodies including type II cryoglobulins with RF activity. Notably, clinical manifestations such as palpable purpura, glomerulonephritis, and peripheral neuropathy are attributed to type II cryoglobulinemia. Previous studies have supported that the lymphocytic inflammatory infiltrate which causes local damage of epithelial structures is rich in T lymphocytes.^1^ On the contrary, the cryoglobulinaemic-associated clinical manifestations are immune complex mediated and, in this case, the inflammatory infiltrate of the salivary glands is dominated by B cells which seems to mature and produce autoantibodies within ectopic germinal centre-like structures. The clinical manifestations of SS can be classified into glandular (oral and eye dryness and parotid swelling), extra-glandular peri-epithelial (primary biliary cirrhosis, interstitial nephritis, bronchiolitis) due to the T cell enriched inflammatory infiltrate, extra-glandular non-specific (arthralgias, arthritis, Raynaud’s phenomenon, fatigue) attributed to non-well defined underlying mechanisms, and extra-glandular extra-epithelial (palpable glomerulonephritis, peripheral neuropathy, purpura), which are type II cryoglobulinaemic mediated due to B cell hyperactivity.^1,4^

Hematologic abnormalities may be also observed in the context of SS, including anaemia, leukopenia, and thrombocytopenia.^5,6^ Leukopenia refers more often to neutrophils (neutropenia), and less commonly to lymphocytes (lymphopenia). Leukopenia has been described in both case reports and large epidemiologic studies^7–12^; it has been also proposed as a risk factor for lymphoma in very few studies.^13–15^ However, the clinical phenotype and the underlying pathogenetic mechanisms of SS-associated leukopenia have not been elucidated. Studies in leukopenic patients with primary immune-deficiencies and systemic lupus erythematosus have shown the presence of anti-neutrophil antibodies and the implication of apoptotic mechanisms in the periphery, as the major pathogenetic mechanisms.^16–19^ More specifically, the role of the cFLIP molecule and the TRAIL pathway of the TNF family have been investigated in lupus patients.^18^ Neutrophils from lupus patients with neutropenia exhibit reduced expression of the anti-apoptotic molecule cFLIP and increased surface expression of the receptors TRAIL 2 and 3 which mediate apoptotic signal into cells. In addition, TRAIL levels are elevated in serum and surface of lymphocytes of neutropenic lupus patients, leading to apoptosis of neutrophils through cell-to-cell interactions. The aims of the current study are: 1) to study the clinical phenotype of SS patients with leukopenia/neutropenia, 2) to detect anti-neutrophil antibodies in SS patients with leukopenia/neutropenia, and 3) to explore pathogenetic mechanisms of leukopenia/neutropenia in SS patients, linked to the TRAIL pathway and the anti-apoptotic molecule cFLIP.

## METHODS

The proposed methodology is as follows:

1. From the HarmonicSS cohort of SS patients who fulfil the 2016 ACR/EULAR and are followed up in the centre of excellence of the Pathophysiology Department, those who present with leukopenia (<4000cells/mm^3^) and neutropenia (1500cells/mm^3^) for at least 1 year and after 3 consecutive measurements 3 months apart, will be identified. All SS leukopenic/neutropenic patients will undergo bone marrow biopsy to exclude myelodysplasia, myelophthisis or any other cause of insufficient bone marrow production or maturation of granulocytes.
2. SS patients without leukopenia/neutropenia will be used as control group, matched according to age, gender, and disease duration in 1:1 ratio with SS leukopenic/neutropenic patients.
3. Healthy controls will be also included, matched according to age and gender in 1:1 ratio with the SS leukopenic/neutropenic patients.
4. Analyses of the clinical phenotype of SS patients with leukopenia/neutropenia and potential associations.
5. Serum and PBMCs will be collected, and subsequently neutrophils and lymphocytes will be isolated from at least 10 individuals in each study group.
6. Anti-neutrophil antibodies will be detected with flow cytometry, as described previously.^17,19^ Initially, a “source of neutrophils” will be generated from a mixture of fresh anticoagulated blood samples of 10 healthy controls and will be treated with chloride ammonium based red blood cell lysing agent; this “source of neutrophils” will then be used for incubating SS patients’ serum with or without leukopenia/neutropenia and healthy controls’ serum, at room temperature for 30 min. Anti-neutrophil antibodies will be detected by FITC conjugated rabbit antibodies against human IgG and IgM with flow cytometry.
7. Measurement of soluble TRAIL in the serum of SS patients and healthy controls (ELISA).
8. Detection of TRAIL 2 and 3 receptors on neutrophils and TRAIL on lymphocytes with flow cytometry in SS patients and healthy controls.
9. In vitro incubation of neutrophils from SS patients and healthy controls and apoptosis studies (TUNNEL staining).
10. Detection of cFLIP expression levels in neutrophils of SS patients and healthy controls (western blot).

The current study has been approved by the Ethics Committee of the National and Kapodistrian University of Athens and is in full compliance with the general data protection regulations (GDPR) of the European Union. All participants have been informed and have signed a written consent.

## RESULTS

The current study is anticipated to: a) define the clinical phenotype of SS patients with leukopenia/neutropenia and the potential clinical associations, b) reveal the pathogenetic mechanisms implicated in peripheral destruction and apoptosis of neutrophils in SS, facilitating the discovery of potential biomarkers for the diagnosis and treatment of hematologic disorders in the context of SS or other systemic autoimmune disorders.

## CONCLUSION

Studies on the clinical phenotype and pathogenetic mechanisms of leukopenic/neutropenic patients with SS is of clinical significance.
